# 

**Published:** 1888-04

**Authors:** 


Vol. IX.] PUBLISHED QUARTERLY AT TWO DOLLARS A YEAR. SINGLE COPIES 60 cts. [No. 2.

LIBRARY INDEX S.G.O.

### THE JOURNAL OF COMPARATIVE MEDICINE AND SURGERY.

EDITED BY

W. A. CONKLIN, Ph. D., D. V. S.,
DIRECTOR OF ZOOLOGICAL GARDENS, NEW YORK CITY.

RUSH SHIPPEN HUIDEKOPER, M. D., Veterinarian (*Alfort*),
DEAN OF VETERINARY DEPARTMENT, UNIVERSITY OF PENNSYLVANIA.

COLLABORATORS.

COMPARATIVE ANATOMY AND PHYSIOLOGY.

PROF. JOSEPH LEIDY, M.D., L.L.D., . University of Penna.
PROF. HENRY C. CHAPMAN, M.D., . Jefferson Med. College.
PROF. E. C. SPITZKA, M.D., . . . . . . . . New York City.
PROF. R. MEADE SMITH, M.D., . . . University of Penna.
PROF. J. T. DUNCAN, M.D., V.S., . Ontario Veterinary College.
PROF. C. M. O'LEARY, M.D., L.L.D., . . . New York City.

COMPARATIVE PATHOLOGY.

HENRY O. MARCY, M.D. . . . . . . . Boston, Mass.
PROF. JAMES TYSON, M.D., . . . . University of Penna.
PROF. R. H. FITZ, M.D., . . . . . . Harvard University.
PROF. WM. OSLER, M.D., . . . . . University of Penna.
E. O. SHAKESPEARE, M.D., . . . . Blockley Hospital, Phila.
A. W. CLEMENT, V. S., . . . . . . . . Montreal Vet. College.
THOMAS B. ROGERS, D. V. S., . . . . University of Penna.

COMPARATIVE SURGERY.

PROF. JAMES LAW, F.R.C.V.S., . . . . Cornell University.
PROF. C. P. LYMAN, F.R.C.V.S., . . . Harvard University.
PROF. D. McEACHRAN, F.R.C.V.S., . . Montreal Vet. College.
PROF. A. T. YOKURA, V.S., . . . Imperial School, Tokio, Japan.
PROF. JAMES L. ROBERTSON, M.D., V.S., Am. Vet. College.
PROF. WILLIAM L. ZUILL, M.D., D.V.S., University of Penna.

A. L. HUMMEL, M. D., PUBLISHER.

8 SPRUCE STREET, NEW YORK, and

No. 117 Filbert Street, Philadelphia, Pa.

LONDON: BALLIERE TINDALL & COX.

